# Association between cerebral blood flow variation and cognitive decline in older patients undergoing hemodialysis

**DOI:** 10.3389/fnagi.2024.1457675

**Published:** 2024-09-17

**Authors:** Yidan Guo, Wei Cui, Pengpeng Ye, Yang Luo

**Affiliations:** ^1^Department of Nephrology, Beijing Shijitan Hospital, Capital Medical University, Beijing, China; ^2^Department of Neurology, Beijing Shijitan Hospital, Capital Medical University, Beijing, China; ^3^Division of Injury Prevention and Mental Health National Center for Chronic and Non-Communicable Disease Control and Prevention, Chinese Center for Disease Control and Prevention, Beijing, China

**Keywords:** hemodialysis, cognitive decline, cerebral arterial mean flow velocity, transcranial Doppler ultrasound, neurocognitive battery

## Abstract

**Background:**

The mechanism of cognitive impairment in hemodialysis patients is multifactorial. The relationship between cerebral blood flow and the decline of cognitive function is poorly understood.

**Objective:**

To investigate the association between cerebral blood flow variation and decline of cognitive function in older patients undergoing hemodialysis.

**Methods:**

In this prospective observational cohort study of 121 older patients undergoing hemodialysis, we used transcranial Doppler ultrasound (TCD) to measure cerebral arterial mean flow velocity (MFV) throughout dialysis, assessed cognitive function at baseline and 12-month follow-up, and then analyzed associations between MFV and changes on cognitive scores.

**Results:**

TCD recordings demonstrated a significant reduction in MFV throughout dialysis, which were significantly correlated with cumulative ultrafiltration volume (rho 0.356, *p* < 0.001), ΔSBP (rho 0.251, *p* = 0.005), and ΔMAP (rho 0.194, *p* = 0.032). Compared with the baseline assessments, cognitive scores of participants at the 12-month follow-up were significantly worsened in global cognition (MOCA), some tests of memory (CFT-memory), executive function (TMT-B, SCWT-C, and SCWT-T), attention/processing speed (SDMT), and visuospatial function (CFT-copy) (*p* < 0.05). The worsening scores in global cognition (MOCA) (*β* = 0.066, 95% CI 0.018–0.113, *p* = 0.007) and some tests of memory (AVLT5) (*β* = 0.050, 95% CI 0.004–0.097, *p* = 0.035) and executive function (TMT-B, SCWT-C, SCWT-T) (*β* = 1.955, 95% CI 0.457–3.453, *p* = 0.011; *β* = 0.298, 95% CI 0.112–0.484, *p* = 0.002 and *β* = 1.371, 95% CI 0.429–2.303, *p* = 0.004, respectively) were significantly associated with the reduction of MFV.

**Conclusion:**

Hemodialysis may significantly reduce cerebral blood flow in older patients; Repetitive intradialytic decreases in CBF may be one of the mechanisms underlying the decline of cognitive function.

**Clinical trial registration:**

https://register.clinicaltrials.gov/prs/app/action/SelectProtocol?sid=S000C5B5&selectaction=Edit&uid=U0003QEL&ts=4&cx=-djoi2

## Introduction

The heavy burden of cognitive impairment in hemodialysis patients has received great attention in the last decade, not only because it has a higher prevalence compared with that in the general population, but also because the decline of cognitive function is independently associated with poor quality of life and clinical adverse outcomes (Drew et al., 2015; Angermann et al., 2018; Cheung and LaMantia, 2019). Currently, the mechanisms of cognitive decline in hemodialysis patients are still not fully elucidated, some studies are focused on the issue of brain ischemia jury which is regarded to be one of the main etiologies of cognitive impairment, besides some common risk factors like older age, lower education level, history of stroke, and hypertension, dialysis-related risk factors like insufficient clearance of renal toxins, prolonged dialysis vintage, micro-inflammation, and hemodynamic instability were reported to be associated with cognitive impairment and its progression in hemodialysis patients (Kurella Tamura et al., 2016; O’Lone et al., 2016; Drew et al., 2019).

As conventional hemodialysis treatment includes the water removal process, it is inevitable for the patients to experience a certain degree of hemodynamic changes (Ma and Zhao, 2017). There is increasing evidence that hemodynamic instability during dialysis treatment might related to brain injury including ischemia stroke and cognitive impairments (Drew and Sarnak, 2014; Teuwafeu et al., 2023). A recent prospective study from the Netherlands applied positron emission tomography-computed tomography (PET-CT) to compare cerebral blood flow (CBF) changes before and during hemodialysis treatment, the results showed that conventional hemodialysis induces a significant reduction in global and regional CBF in older patients during hemodialysis, and repetitive intradialytic decreases in CBF may be one of the mechanisms by which hemodialysis induces cerebral ischemic injury (Polinder-Bos et al., 2018). This result provided a hint about the influence of CBF reduction on the decline of cognitive function in hemodialysis patients. However, performing the PET-CT examination during dialysis treatment is too complex in practical settings. Hence, finding alternative measures to reflect the variation in brain blood volume in hemodialysis patients is necessary.

Transcranial Doppler (TCD) ultrasound is a noninvasive, convenient tool that is capable of performing repeated measurements of cerebral blood flow throughout a hemodialysis session, it has been widely applied in the evaluation of CBF (Stefanidis et al., 2005; Shaker et al., 2018). Depending on the previously reported features of hemodynamic changes in dialysis patients and the current understanding of the relationship between brain ischemia and cognitive decline, we hypothesized that TCD-measured cerebral blood flow would be reduced during hemodialysis sessions and these repetitive changes of CBF would be associated with the progressive cognitive impairment. We validated our hypotheses in a prospective cohort, our study aimed to explore whether there was cerebral blood flow reduction in dialysis sessions and whether these changes were associated with the decline of cognitive function over a 12-month follow-up.

## Materials and methods

### Study design and participants

This is a prospective observational cohort study. We measured cerebral arterial mean flow velocity (MFV), an index of brain blood flow, at several time points throughout a dialysis session with TCD. Using well-validated neuropsychological batteries, we compared cognitive function at baseline and 12 months of follow-up and accessed correlations between the variation of MFV and changes in cognitive function scores. We recruited eligible patients from the hemodialysis centers of Beijing Shijitan Hospital from April to June 2022. The inclusion criteria were as follows: (1) age ≥ 50 years old, (2) end-stage kidney disease with maintained hemodialysis treatment for a minimum of 3 months, (3) willing to join the study and provide written informed consent, and (4) the patient’s native language was Chinese. The exclusion criteria were as follows: (1) unable to complete the middle cerebral artery ultrasound examination, (2) unable to complete a 90-min cognitive and physical function battery for reasons such as sensory (e.g., visual and hearing) or motor impairment, (3) experienced disturbance of consciousness or recently diagnosed with psychosis, and (4) had a plan of kidney transplantation within 12 months of baseline. This study was approved after ethical review by the Institutional Ethical Review Board of Beijing Shijitan Hospital, Capital Medical University (Approval No. sjtkyll-lx-2022-078), and registered on Clinicaltrials.gov under identifier NCT05555836. Written informed consent was completed for each participant.

### Clinical variables

Patients’ sociodemographic information and basic characteristics were obtained from their medical charts at the time of enrollment. Medical history, including history of cardiovascular disease (CVD, a composite of either coronary artery disease and/or peripheral vascular disease), stroke, diabetes, hypertension, smoking, alcohol intake, and dialysis vintage, was defined by the patient’s history or documentation in the patient’s medical chart. Physical examination included mean monthly systolic blood pressure (SBP), diastolic blood pressure (DBP), and body mass index (BMI). Pre-dialysis blood tests included measurement of the serum levels of hemoglobin, albumin, calcium, phosphate, intact parathyroid hormone (iPTH), and C-reactive protein (CRP). The estimated glomerular filtration rate (eGFR) was calculated by using the Chronic Kidney Disease Epidemiology Collaboration (CKD-EPI) creatinine equation with an adjusted coefficient of 1.1 for the Asian population, the single-pool Kt/V was calculated from the pre-and post-dialysis serum urea nitrogen levels as we applied in the previous study (Guo et al., 2022).

### TCD monitored cerebral arterial mean flow velocity in hemodialysis sessions

All participants underwent their routine prescribed dialysis treatment. The median blood flow rates were 280 (IQR, 240.0–320.0) mL/min. Dialysate temperature was set at 36.5°C. On the day of dialysis, the patient was in a supine position. We used transcranial Doppler ultrasound to monitor the mean flow velocity (MFV) of the middle cerebral artery (MCA) throughout dialysis. The average MCA blood flow velocity was recorded at seven time points (T_1_ to T_7_), including 15 min before dialysis, 15, 30, 60, 120, 180 min during dialysis, and after dialysis. Meanwhile, we also recorded dialysis-related variables, including SBP, DBP, and ultrafiltration volume. To minimize measurement errors, all Doppler readings were performed by two trained examiners following standardized protocols.

### Assessment of cognitive function

Cognitive assessments were conducted at study enrollment and repeated at 12-month follow-up. According to the recommendation of the fifth version of the Diagnostic and Statistical Manual of Mental Disorders (DSM-V), we evaluated the global cognitive function with the Beijing version of the Montreal Cognitive Assessment (MOCA), and cognitive function in five different cognitive domains (verbal and memory, complex attention, executive function, language, visual–spatial function), each domain was assessed using validated neuropsychological tests (Sachdev et al., 2014; Tian et al., 2020). It included: (1) attention/processing speed, using the Symbol Digit Modalities Test (SDMT), and the Chinese-modified version of the Trail Making Test A (TMT-A) (Eadie and Shum, 1995; Ma et al., 2015); (2) executive function, using the Chinese-modified version of the Trail Making Tests B (TMT-B), and a modified version of the Stroop Color-Word Test (SCWT) (Zhou and Jia, 2009); (3) verbal memory, using the Chinese version of the Auditory Verbal Learning Test (AVLT) for short-and long-delay free recall and complex figure for visual memory (delayed recall test; Chinese version) (Dai et al., 1990); (4) language, using the Chinese-modified versions of Boston Naming Test (BNT) and Animal Fluency Test (AFT) (Guo et al., 2009; Huang et al., 2016); and (5) visual-space function, using the Rey–Osterrieth Complex Figure (CFT) (Li et al., 2018). All of the staff for the neuropsychological assessments were trained and certified by the same neuropsychologist before the study commencement. To avoid the influence of hemodynamic changes during the dialysis treatment, a neuropsychological evaluation was conducted individually on the day after a dialysis session. Patients were kept comfortable during their cognitive assessments, including rest periods if needed.

### Statistical analysis

Baseline characteristics are presented in descriptive statistics, with mean and standard deviation (SD) or median with interquartile range (IQR) given for continuous variables, and percentages given for categorical variables. MFV at each time point throughout dialysis was analyzed using a paired sample Wilcoxon signed-rank test. ΔMFV = MFV (T_1_) − MFV (T_7_). Spearman correlation analysis was performed to evaluate the association between the decline in MFV and dialysis-related variables. Cognitive scores at baseline and 12 months are also paired data and were compared using the Wilcoxon signed-rank test. The correlation between changes in cognitive scores and the percentage of decline in MFV was assessed using Spearman rank correlation.

We used multivariable linear regression models to analyze the relationship between the changes in MFV and worsening cognitive function, with worsening cognitive scores as the dependent variable, percentage of changes in MFV as independent variables, and variables associated with worsening cognition on unadjusted analyses with *p ≤* 0.10 and potential clinical risk factors for cognitive impairment were entered into the multivariate linear regression models as covariates. All analyses were performed with SPSS version 21.0 statistical software (SPSS Inc., Chicago, IL, USA). Statistical significance was set at a value of *p* < 0.05.

## Results

### Baseline characteristics of participants

Among the 140 eligible patients, 12 patients could not complete comprehensive cognitive tests and seven patients’ middle cerebral arteries could not be detected with the TCD, a total of 19 patients were excluded. Finally, 121 patients were enrolled and finished the follow-up. Baseline characteristics of the remaining 121 patients were shown in Table 1, the mean age was 63.63 ± 8.44 years, 19.5% were women, and only 5.8% had less than 6 years of education. Hypertension (97.6%), diabetes (68.3%), and CVD (65.9%) were the most common diseases in medical history. The dialyzers with polyacrylonitrile, polysulfone, and polycarbonate membranes used for hemodialysis were 65.5, 23.4, and 11.1%, respectively. The average weekly hemodialysis treatment length was 11.40 ± 0.78 h.

**Table 1:** Demographic and clinical characteristics of participants at baseline.

Variables	Mean (SD) or median (IQR) or *n* (%)
Sample size	121
Age (years)	63.63 ± 8.44
Gender, male	99 (80.5%)
Education level	
<6 years	7 (5.8%)
6–12 years	80 (66.1%)
>12 years	34 (28.1%)
Smoking history	42 (34.1%)
Alcohol intake	21 (17.1%)
Medical history	
Diabetes	84 (68.3%)
Hypertension	120 (97.6%)
Stroke	30 (24.4%)
CVD	81 (65.9%)
Dialysis vintage, mo.	57.00 (24.00, 101.50)
Single-pool Kt/V	1.21 ± 0.21
BMI, kg/m^2^	23.03 (21.00,26.10)
SBP (mmol/L)	155.00 (143.00, 179.00)
DBP (mmol/L)	78.00 (72.00, 88.00)
Hb (g/L)	113.58 ± 12.73
Alb (g/L)	36.46 ± 3.46
TC (mmol/L)	3.44 ± 0.81
TG (mmol/L)	1.50 ± 0.86
Calcium (mmol/L)	2.16 ± 0.16
Phosphate (mmol/L)	1.86 ± 0.58
iPTH (pg/mL)	188.20 (101.00, 279.20)
CRP (mg/L)	6.46 ± 8.17
eGFR (mL/min·1.73 m^2^)	9.86 ± 3.25

Data were presented as mean ± SD or median (interquartile range) for continuous variables and number (%) for categorical variables. CVD, cardiovascular disease; Kt/V, an indicator for evaluating dialysis adequacy; BMI, body mass index; SBP, systolic blood pressure; DBP, diastolic blood pressure; Hb, hemoglobin; ALB, albumin; TC, total cholesterol; TG, triglyceride; iPTH, intact parathyroid hormone; CRP, C-reactive protein; eGFR, estimated glomerular filtration rate.

### Cerebral blood flow changes in a hemodialysis session

**Figure 1:**
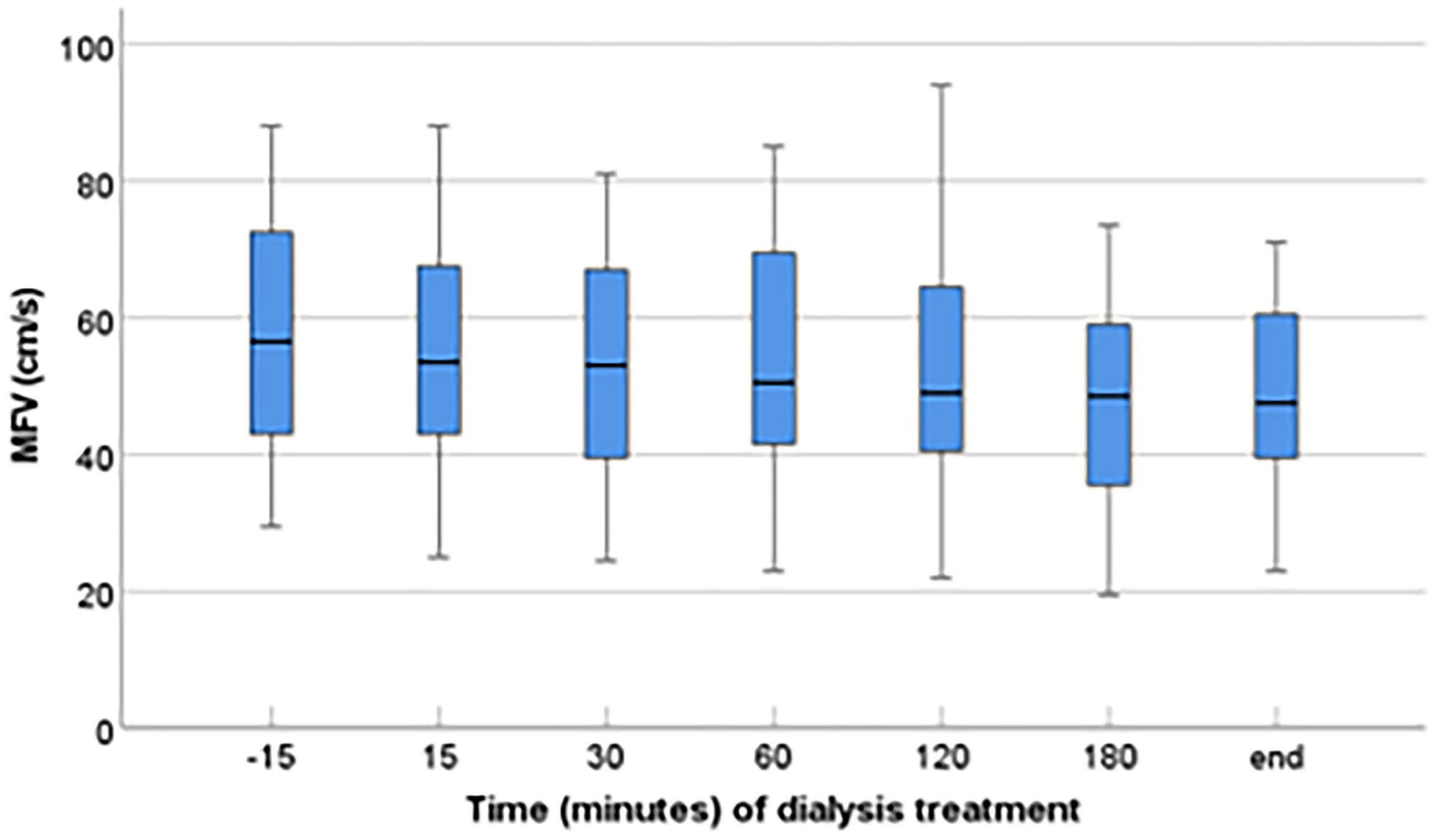
Variation of cerebral mean flow velocity (MFV) of the middle cerebral artery during hemodialysis session. The change in MFV during dialysis sessions is presented as a median value, IQR, maximum, and minimum values. TCD recordings were taken before and during dialysis, demonstrating a significant decline in MFV during dialysis (*p* < 0.001).

The readings of MFV and other dialysis-related indicators (ultrafiltration volume, SBP, DBP, and MAP) before and during dialysis are shown in Table 2. MFV generally reduced after dialysis began, remaining lower up to the completion of dialysis (median MFV reading from 56.5 to 47.5 cm/s; *p* < 0.001). The median percentage of decline was 14.00% (IQR, 2.47–23.81%) (Figure 1; Table 2). Spearman correlation analysis results show that cumulative ultrafiltration volume (rho 0.356, *p* < 0.001), ΔSBP (rho 0.251, *p* = 0.005), ΔMAP (rho 0.194, *p* = 0.032), were significantly correlated with ΔMFV.

**Table 2:** Variation in MFV and other clinical variables before and during hemodialysis.

Variables	Data before and during dialysis (min)							Differences (T_1_ vs. T_7_)			
	T_1_ −15	T _2_ 15	T_3_ 30	T_4_ 60	T_5_ 120	T_6_ 180	T_7_ Completion	Absolute change (Δ)	*p*-value	Percentage change (%)	*p*-value
Ultrafiltration (L)	0 (0, 0)	0.14 (0.08, 0.16)	0.28 (0.15, 0.33)	0.55 (0.30, 0.65)	1.10 (0.60, 1.30)	1.65 (0.90, 1.95)	2.20 (1.80, 2.80)	2.20 (1.80, 2.80)	<0.001	–	–
SBP (mmHg)	155 (143, 179)	145 (134, 160)	149 (139, 161)	149 (138, 163)	157 (145, 169)	154 (142, 168)	166 (119, 180)	−9 (−18, 2)	<0.001	−6.82 (−11.41, 1.10)	<0.001
DBP (mmHg)	78 (72, 88)	78 (70, 84)	80 (71, 84)	81 (72, 86)	82 (71, 87)	82 (72, 90)	85 (77, 93)	−6 (−13, 0)	<0.001	−8.33 (−17.81, 0)	<0.001
MAP (mmHg)	103.33 (95.33, 114.33)	100.67 (96.67, 109.33)	101.67 (94.67, 107.67)	103.33 (94.00, 110.67)	106.00 (99.00, 111.33)	106.00 (97.33, 113.67)	110.00 (104.00, 123.00)	−8 (−12, 1.33)	<0.001	−7.95 (−12.24, 1.19)	<0.001
MFV (m/s)	56.5 (43, 72.5)	53.5 (43, 67.5)	53 (39.5, 67)	50.5 (41.5, 69.5)	49 (40.5, 64.5)	48.5 (35.5, 59.0)	47.5 (39.5, 60.5)	7 (1, 15)	<0.001	14.00 (2.47, 23.81)	<0.001

Absolute change (Δ) = T_1_ value − T_7_ value, Percentage change (%) = (T_1_ value − T_7_ value)/T_1_ value × 100.

### Cognitive function at baseline and 12 months follow-up

During the follow-up, four participants withdrew, and 13 patients died. Paired baseline and 12-month cognition assessments were possible in 104 participants. The raw scores of each test are presented in Table 3. Compared with the baseline assessments, participants’ cognitive scores at 12-month follow-up were significantly worsened in global cognition (MOCA), one test of memory (CFT-memory), three tests of executive function (TMT-B, SCWT-C, and SCWT-T), one test of attention/processing speed (SDMT), and one test of visuospatial function (CFT-copy). The worsening scores in global cognition (MOCA) and three tests of executive function (TMT-B, SCWT-C, and SCWT-T) were significantly correlated with the percentage of decline in MFV (*p* < 0.05) (Table 3).

**Table 3:** Differences in cognitive scores between baseline and 12-month follow-up and their correlation with percentage decrease in MFV.

Domains	Tests	Raw-scores (*n* = 104)			Correlation with percentage of decline in MFV	
		Baseline	12-month	*p*-value	*Rho*	*p*-value
Global cognition	MoCA-BJ	24.77 ± 3.34	24.14 ± 3.03	0.005	0.169	0.048
Memory	AVLT 5	4.79 ± 2.98	4.66 ± 2.95	0.314	0.150	0.128
	AVLT1-5	26.47 ± 10.40	25.70 ± 11.42	0.074	0.051	0.604
	CFT-memory	13.68 ± 6.85	11.75 ± 5.96	<0.001	0.141	0.152
Executive function	TMT-B	170.57 ± 61.71	186.02 ± 68.32	<0.001	0.364	<0.001
	SCWT-C	46.08 ± 4.75	44.68 ± 4.31	0.003	0.212	0.031
	SCWT-T	80.23 ± 26.74	87.74 ± 31.63	0.002	0.305	0.002
Attention/processing speed	SDMT	28.98 ± 11.67	21.08 ± 8.79	<0.001	0.105	0.287
	TMT-A	79.96 ± 32.65	77.49 ± 32.48	0.322	0.061	0.538
Language	BNT	29.02 ± 1.41	29.00 ± 1.20	0.820	0.009	0.926
	AFT	55.92 ± 12.61	54.45 ± 15.18	0.089	0.072	0.465
Visuospatial function	CFT-copy	30.44 ± 3.79	26.25 ± 7.49	<0.001	0.042	0.671

MoCA-BJ, the Chinese Beijing version of the Montreal Cognitive Assessment; AVLT, Auditory Verbal Learning Test; CFT, Rey-Osterrieth Complex Figure; SDMT, Symbol Digit Modalities Test; TMT-A, Trail Making Test A; TMT-B, Trail Making Test B; SCWT, Stroop Color-Word Test; AFT, Animal Fluency Test; BNT, Boston Naming Test.

### Association between the variation of MFV and the cognition decline

Multivariable linear regression analysis showed that the worsening scores in global cognition (MOCA) (*β* = 0.066, 95% CI 0.018–0.113, *p* = 0.007), one tests of memory (AVLT5) (*β* = 0.050, 95% CI 0.004–0.097, *p* = 0.035) and all three tests of executive function (TMT-B, SCWT-C, SCWT-T) (*β* = 1.955, 95% CI 0.457–3.453, *p* = 0.011; *β* = 0.298, 95% CI 0.112–0.484, *p* = 0.002 and *β* = 1.371, 95% CI 0.429–2.303, *p* = 0.004, respectively) were significantly associated with the percentage of decline in MFV (Table 4).

**Table 4:** Multivariable linear regression analysis for association between the worsening cognitive scores and percentage of reduction of MFV.

Domains	Tests	Unstandardized coefficients			*Beta*	*t*	*p*
		*B*	*95% CI for B*	*Std. Error*			
Global cognition	MoCA-BJ	0.066	0.018, 0.113	0.024	0.447	2.757	0.007
Memory	AVLT 5	0.050	0.004, 0.097	0.023	0.342	2.148	0.035
	AVLT1-5	0.219	−0.233, 0.671	0.227	0.135	0.964	0.338
	CFT-memory	0.134	−0.048, 0.316	0.091	0.249	1.465	0.147
Executive function	TMT-B	1.955	0.457, 3.453	0.753	0.448	2.595	0.011
	SCWT-C	0.298	0.112, 0.484	0.093	0.542	3.193	0.002
	SCWT-T	1.371	0.439, 2.303	0.469	0.491	2.924	0.004
Attention/Processing Speed	SDMT	0.085	−0.108, 0.278	0.097	0.105	0.876	0.384
	TMT-A	0.264	−0.807, 1.335	0.539	0.089	0.490	0.625
Language	BNT	0.005	−0.031, 0.041	0.018	0.047	0.262	0.794
	AFT	0.081	−0.290, 0.451	0.186	0.079	0.432	0.667
Visuospatial function	CFT-copy	0.120	0.000, 0.240	0.060	0.251	1.987	0.050

Models were adjusted for age, sex, education level, smoking history, alcohol intake, medical history, hemodialysis vintage, Kt/V, BMI, SBP, DBP, and the serum level of Hb, ALB, CRP, TC, TG, calcium, phosphate, and iPTH.

## Discussion

In this prospective study, we demonstrated cerebral blood flow kept reducing in a hemodialysis session, the median percentage of this reduction was 14%. This brain hemodynamic change was closely correlated with the decline of global cognitive function, notably, the association between cerebral blood flow reduction and the cognitive decline appears to be inconsistent in different cognitive domains, only the decline of the executive domain and memory domain was found to be related to cerebral blood flow reduction. Our data validated the impact of hemodialysis-related reduction of brain blood perfusion on the progression of cognitive impairment among hemodialysis patients.

Previous studies indicated that patients undergoing hemodialysis not only have a high prevalence of cognitive impairment compared to the general population, but also experience a decline of cognitive function over time, and this is predominantly among older individuals (Polinder-Bos et al., 2018; Luo et al., 2020; Guo et al., 2022). It seems urgent to explore the potentially related risk factors and etiologies of cognitive decline. Currently, brain ischemia has been confirmed as one of the main etiologies for the pathological process of cognitive impairment, and finding brain ischemia-related risk factors has become a hot topic in this area (Romero et al., 2009; Igari et al., 2019). Older age, atherosclerosis-related factors like diabetes, hyperlipidemia, and hypertension are confirmed to be related to the impairments of cognitive function. These risk factors were also validated among hemodialysis patients with cognitive impairments. Notably, some dialysis-related risk factors like insufficient clearance of renal toxins, prolonged dialysis vintage, and hemodynamic instability were also reported to be associated with cognitive impairment and its progression in hemodialysis patients (MacEwen et al., 2017; Moist and McIntyre, 2019). However, the characteristics of the hemodynamic changes in hemodialysis patients are not fully elucidated. Polinder-Bos et al. (2018) applied [^15^O] H_2_O PET-CT to compare CBF changes before and during hemodialysis treatment, the results showed that conventional hemodialysis induces a significant reduction in global and regional CBF in older patients during hemodialysis. However, performing the [^15^O] H_2_O PET-CT examination during dialysis treatment is not convenient in practical settings, so finding alternative measures to reflect the variation in brain blood volume in hemodialysis patients is necessary. TCD ultrasound is a noninvasive, convenient tool that is capable of performing repeated measurements of cerebral blood flow throughout a hemodialysis session, it has been widely applied in the evaluation of CBF (Stefanidis et al., 2005; Ghoshal and Freedman, 2019). In our study, we measured MFV at different time points in a dialysis session, and there was an average of 14% reduction in MFV throughout dialysis, which was significantly correlated with cumulative ultrafiltration volume, and changes in blood pressure. Richerson et al. (2022) also found significant relationships between the variations of cerebral oxygenation saturation and intradialytic blood pressure change and cumulative ultrafiltration dose in 26 hemodialysis patients. Our results indicated that there is a brain blood flow reduction during hemodialysis treatment, this status might lead to brain ischemia in combination with other conventional risk factors like older age, and vascular atherosclerosis-related factors.

Another major issue in building up the relationship between brain ischemia and cognitive decline is identifying the progression features of cognitive impairment with the prolonged dialysis vintage and associated risk factors. Drew et al. (2017) demonstrated significant cognitive decline over time, particularly within tests of executive function in 314 prevalent hemodialysis patients. Older age was the only statistically significant risk factor for steeper cognitive decline, as for other potential related risk factors, the authors believed that there were 156 deaths in the 2 years of follow-up, this high mortality rate might have some influence in identifying the potential risk factors in their study. Findlay et al. (2019) reported that patients undergoing hemodialysis experienced a transient decline in cerebral blood flow using the measurement of MFV with TCD, and this reduction of brain blood flow was correlated with intradialytic cognitive decline. Compared with those studies, we tested these related issues in a group of 121 hemodialysis patients, the results of our study indicated that the worsening scores were in the test of global cognition, executive function, and some tests of memory function, the inconsistent decline of cognitive function in different domains also indicated the ischemia related injury in various parts of the brain is different. Taken together, our data provide evidence that both reduction of CBF during dialysis sessions and decline of cognitive function exist in hemodialysis patients, and this reduction of brain blood flow may play a pivotal role in the progression of cognitive impairment in hemodialysis patients. Strictly administrating the water intake between hemodialysis sessions and appropriately setting a proper ultrafiltration rate during dialysis will be helpful in keeping the hemodynamics stable during hemodialysis, which in turn retard the progression of brain ischemia-related cognitive impairment.

We demonstrated the relationship between the reduction of brain blood flow and cognitive decline in hemodialysis patients in this prospective cohort. However, we recognize the following limitations. As an observational study, we do not have data for patients who are younger than 50 because the assessment batteries were all prepared for middle and older aged populations, at the same time, some senior-aged patients who have visual and hearing impairments could not be enrolled in our study, all these could bring selection bias in our study. We also need to clarify that TCD measurement is an indirect measure of cerebral blood flow, the accuracy of the TCD measurement is inferior to the [^15^O] H_2_O PET-CT examination, which is regarded as a gold standard in this area; the MFV results may be influenced by some unaccounted variables like blood viscosity and blood vessel diameter, which we did not have records in this study.

In conclusion, there is a significant reduction in cerebral blood flow in older patients undergoing Hemodialysis, which is related to cumulative ultrafiltration volume and changes in blood pressure. This repetitive intradialytic decrease in cerebral blood flow may be one of the major mechanisms underlying the decline of cognitive function in hemodialysis patients.

## Data Availability

The original contributions presented in the study are included in the article/supplementary material, further inquiries can be directed to the corresponding author.
